# Spatial analysis of measles vaccination coverage in the State of São Paulo

**DOI:** 10.1186/s12889-022-14797-z

**Published:** 2023-01-05

**Authors:** Ysabely de Aguiar Pontes Pamplona, Anderson Marcos Vieira do Nascimento, Ricardo Alves de Olinda, Carolina Luisa Alves Barbieri, Alfésio Luís Ferreira Braga, Lourdes Conceição Martins

**Affiliations:** 1 Catholic University of Santos (Universidade Católica de Santos – Programa de Pós-Graduação strictu senso em Saúde Coletiva), Av. Conselheiro Nebias, 300, Sala 106, São Paulo, Santos CEP: 11.015-002 Brazil; 2 Oswaldo Cruz Foundation, Rio de Janeiro, Brazil; 3 State University of Paraíba, Campina Grande, Paraíba Brazil

**Keywords:** Vaccine coverage, Spatial analysis, Global moran’s index, Local moran’s index, Mixed ecological study, Secondary data

## Abstract

**Background:**

Measles is a contagious viral disease that seriously affects children. The measles vaccine is widely recommended in Brazil and in the world; however, the disease remains relevant for the health authorities. The aim of the present study was to evaluate first and second dose of measles vaccine coverage (VC) in the cities of São Paulo and its spatial dynamics between 2015 and 2020. Method: In this mixed-type ecological study, we used secondary, public domain data from 2015 to 2020, extracted from the Digital Information System of the National Immunization Program, Mortality Information System and the National Live Birth Information System. After calculating the VC, the following four categories were created: very low, low, adequate, and high, and the spatial autocorrelation of VC was analyzed using the Global and Local Moran’s statistics.

**Results:**

A steady decline in adherence to the vaccination was observed, which dynamically worsened until 2020, with a high number of cities fitting the classification of ineffective coverage and being potentially harmful to the effectiveness of the immunization activities of their neighbors.

**Conclusion:**

A direct neighborhood pattern was observed between the units with low vaccination coverage, which implied that the reduction in measles VC was somehow related to and negatively influenced by the geographic location and social culture of these areas.

## Background

Measles is a contagious viral disease that seriously affects children; however, people of any age can be infected, especially immunosuppressed individuals or those with compromised nutritional status [1].

During the 1960s and 1970s, measles was the leading cause of death among children younger than 4 years in Brazil, and this created the need to develop strategies to address this health problem, which predominantly affected socially vulnerable populations; however, the disease continued to spread universally [2]. These measures eventually developed through the implementation of vaccination against the disease, which was introduced in 1963, and through the mandatory notification of all suspected cases of the disease in Brazil, in order to allow health authorities to have real data related to the epidemiological course of the disease [3].

Since then, the measles vaccine has been widely recommended in Brazil and in the world; however, the disease continues to be a cause for concern for the health authorities as it is one of the main causes of death in children younger than 5 years, with 89,780 related deaths worldwide in 2016 [4].

The vaccination agenda includes the availability of a range of vaccines, including the triple viral vaccine, a combined immunization against measles, mumps, and rubella (MMR), with a basic regimen of two doses [4, 5]. In Brazil, vaccines that are considered essential are distributed and administered free of charge in services operated by the Unified Health System (SUS), through the National Immunization Program (PNI), and the measles vaccine has been administered since the 1960s [6].

In 2016, Brazil received the certificate of elimination of the circulation of the measles virus of the World Health Organization, with this result being associated with a good vaccination coverage; however, in 2019, new outbreaks of the disease were reported across the country, with 20,901 confirmed new cases, which are directly linked to the decline in the vaccination coverage of children younger than 5 years [6]. The Ministry of Health (MS) reported approximately 30,037 confirmed cases of the disease between 2019 and 2021**,** a number that is a cause for concern for the health authorities [1].

Although the measles vaccine has been shown to be effective and safe, specialists claim that failures are directly associated with disparities in the vaccination coverage process, as evidenced by the high incidence of measles cases and deaths even in high-income countries [7–9].

In 2017, the Ministry of Health conducted a study with the objective of identifying the reasons why vaccination coverage was steadily declining and gave nine reasons to explain the drop in the numbers of vaccinated individuals as follows: the misleading perception that if the disease is not circulating in society then vaccination is not needed; news that discredit vaccines, mainly due to the strong national and international anti-vaccination movements; deficiency in the operation of the Health Information Systems; and incompleteness of the vaccination scheme, justified by a feeling of protection with a single dose, which confirms that the incompleteness of the vaccination program is a health problem at the national level [10].

Currently, Brazil faces an urgent need to improve adherence to childhood immunization, which, after remaining at high levels for more than two decades, has shown a concerning decline, with the lowest levels of childhood vaccination coverage observed in 2017, including the vaccines for measles and 17 other diseases [11].

From 2020 and 2021, vaccination rates continued to decline, this time influenced by the excessive workload of healthcare workers caused by the COVID-19 pandemic and the series of restrictions imposed to contain the spread of the disease. Experts say that, during this period, childhood vaccination was reduced by at least 65% and approximately 900,000 children were not vaccinated in the country in 2021 [11, 12].

Given the measles scenario in Brazil, linked to the fall in vaccine coverage (VC), it is essential to think of strategies to face and overcome these difficulties, considering that the immunization process is a strong ally for the control of infectious diseases and for reducing childhood mortality from vaccine-preventable diseases. Therefore, the objective of the study was to evaluate first and second dose measles VC in the cities of São Paulo and its spatial dynamics, between 2015 and 2020.

## Methods

In this mixed ecological study of spatial analysis, we used secondary data obtained from the Database of the IT Department of the Unified Health System (DATASUS)/Health Information (TABNET) for each of the 645 cities in the State of São Paulo in 2015–2020. Owing to the fact that this was a study with secondary data, and according to Resolution No. 510/2016 of the National Health Council, it did not require submission to the research ethics committees.

This research is part of a broader project titled Spatial Analysis of Children’s Vaccine Coverage and its Relationship with Socioeconomic and Health Characteristics in Brazil, funded by the Bill and Melinda Gates Foundation and the Brazilian National Council for Scientific and Technological Development (CNPq).

The following information was obtained: doses administered (from the Immunization System—SI-PNI), live births (from the Live Births System—SINASC), and infant mortality (from the mortality system—SIM). The digital cartograms were obtained from the Brazilian Institute of Geography and Statistics (IBGE).

The following equation was used to calculate measles VC for each city:

$$\boldsymbol{Vaccination}\; \boldsymbol{coverage}\; \boldsymbol{in}\;1\;\boldsymbol{year}\; \boldsymbol{old}\; \boldsymbol{child}=\frac{\boldsymbol{Applied}\; \boldsymbol{doses}\; \boldsymbol{of}\; \boldsymbol{a}\; \boldsymbol{vaccine}\; \boldsymbol{in}\; \boldsymbol{a}\; \boldsymbol{given}\; \boldsymbol{municipality}\; \boldsymbol{and}\; \boldsymbol{year}\dagger}{\boldsymbol{Live}\; \boldsymbol{births}\; \boldsymbol{minus}\; \boldsymbol{deaths}\; \boldsymbol{in}\; \boldsymbol{children}\; \boldsymbol{under}\;1\;\boldsymbol{year}\; \boldsymbol{of}\;\boldsymbol{age}\; \boldsymbol{in}\; \boldsymbol{that}\; \boldsymbol{municipality}\; \boldsymbol{in}\; \boldsymbol{the}\; \boldsymbol{previous}\; \boldsymbol{year}}\ast100$$

†The numerator of the 1st dose is the sum of the first doses of measles, mumps and rubella vaccines plus the viral quadruple vaccine. The numerator of the 2nd dose is the sum of the second doses of measles, mumps and rubella vaccines plus the viral quadruple and the first dose of the tetraviral vaccine.

The VC was stratified according to Braz et al., [13] as follows: very low (0–50), low (50–95), adequate (95–120), and high (≥ 120).

To perform the spatial analysis, we considered the cities with a VC above zero and the cities with first-order neighbors, with the archipelago of Ilha Bela being excluded because it is the only city that does not have neighbors.

The GeoDa software version 1.14 was used to analyze the spatial distribution of VC by city and to check whether it occurred randomly in space or whether the events in one city influenced the events in the neighboring cities. A neighborhood matrix was created to test the spatial autocorrelation between VCs. Subsequently, the following criterion was used for the construction of the matrix: queen contiguity based on first-order neighbor weight.

The Global Moran’s Index (GMI) was used to measure spatial autocorrelation and the Local Moran’s Index (local indicators of spatial association-LISA) was used to analyze the pattern of spatial distribution and the intensity of clusters to analyze the 643 cities in São Paulo. A significance level of 95% and 999 random permutations were considered for both analyses.

The GMI measures the relationship between observations with spatial proximity, providing a single measurement for all cities, with a value ranging from − 1 to + 1. The closer the value is to + 1, the more similar they are, and the closer the value is to − 1, the less auto-correlated the analyzed variable is [14–16].

The occurrence of spatial clusters was evaluated using a Moran map and the significance pattern of these clusters was determined using a LISA map. The result of the Moran map analysis is divided into the following five categories: not significant, showing the municipalities that did not have statistical significance; high-high, showing the cities with high VC, surrounded by other cities with high VC; low-low, showing the cities with low VC, surrounded by cities with low VC; high-low, showing the cities with high coverage, surrounded by cities with low coverage; low–high, showing the cities with low coverage, surrounded by cities with high coverage. The LISA map presents two categories of interpretation as follows: not significant, showing the cities that did not have statistical significance, and significant, showing the cities that had a significance of 95%, 99%, and 99.9% [15, 17–19].

Finally, thematic maps were prepared to better understand and visualize the obtained results using the QGIS 3.10 software.

## Results

**Fig. 1:**
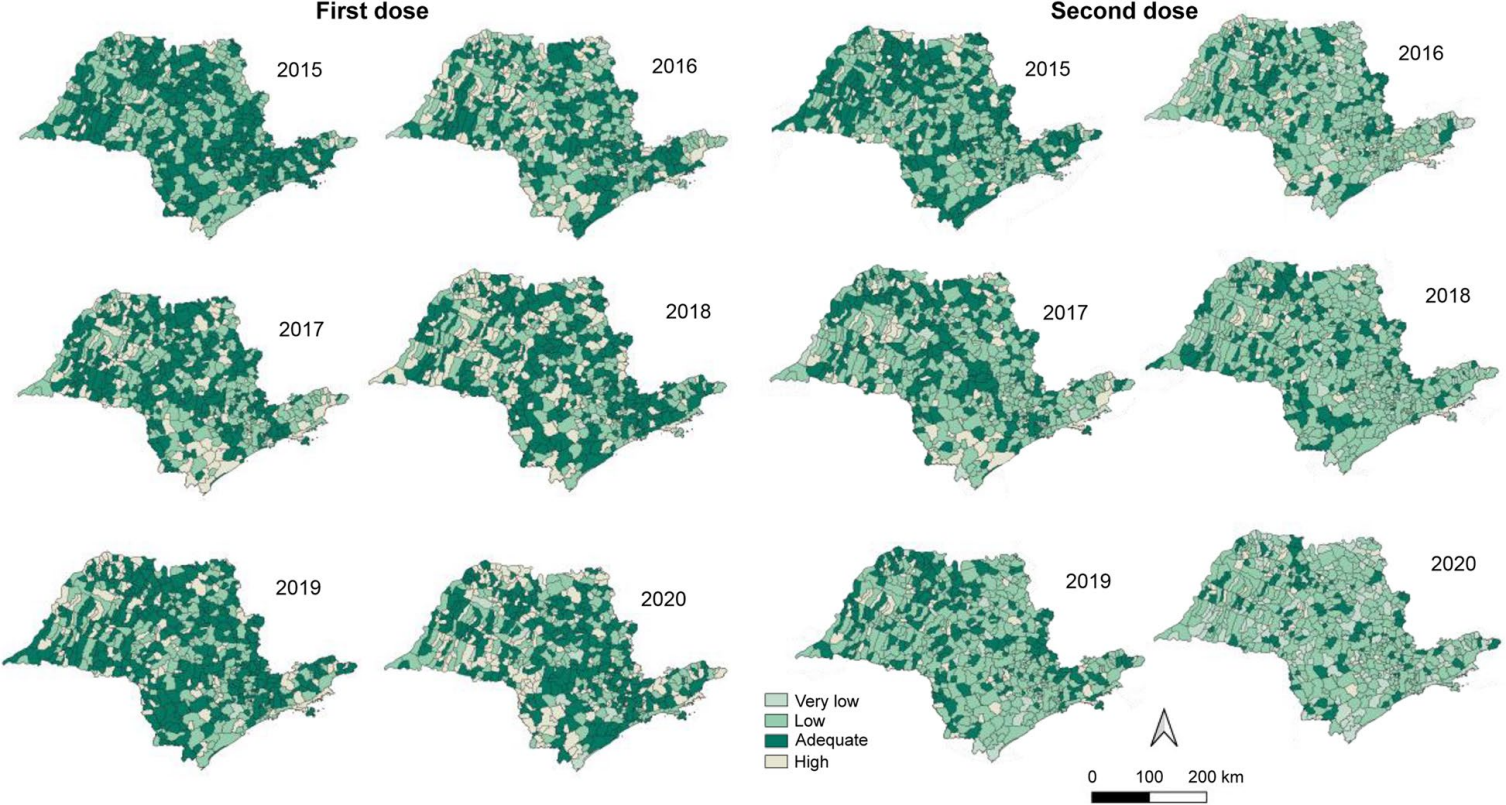
Spatial distribution of first and second dose measles vaccine coverage in the cities of São Paulo between 2015 and 2020

The patterns of VC from 2015 to 2020 were evaluated for the first and second dose of the MMR vaccine (triple viral vaccine) and there was a variability in the pattern of immunization coverage among the years; despite the good vaccination rates in most polygons, there were persistent areas with low or very low coverage = 50% and/or < 50% (Fig. 1).

In the Global Moran’s Index (GMI) analysis, there are values that indicate a significant (*p* < 0.05) and positive spatial autocorrelation between the municipalities of São Paulo for the first dose in the years 2015, 2017, 2018, 2019 and 2020 and for the second dose were in the years 2017, 2018, 2019 and 2020. It should be noted that the autocorrelation analysis for the first dose of the year 2016 and the second dose of the years 2015 and 2016 did not show significant results (Table 1).

**Table 1:** Global Moran’s Index per year based on total vaccination coverage from 2015 to 2020

Dose 1	Global Moran’s I	*p* value	Dose 2	Global Moran’s I	*p* value
2015	0.042	0.043	2015	0.036	0.058
2016	0.038	0.055	2016	0.025	0.118
2017	0.077	0.002	2017	0.101	0.001
2018	0.111	0.001	2018	0.097	0.001
2019	0.094	0.002	2019	0.113	0.001
2020	0.057	0.008	2020	0.106	0.001

**Fig. 2:**
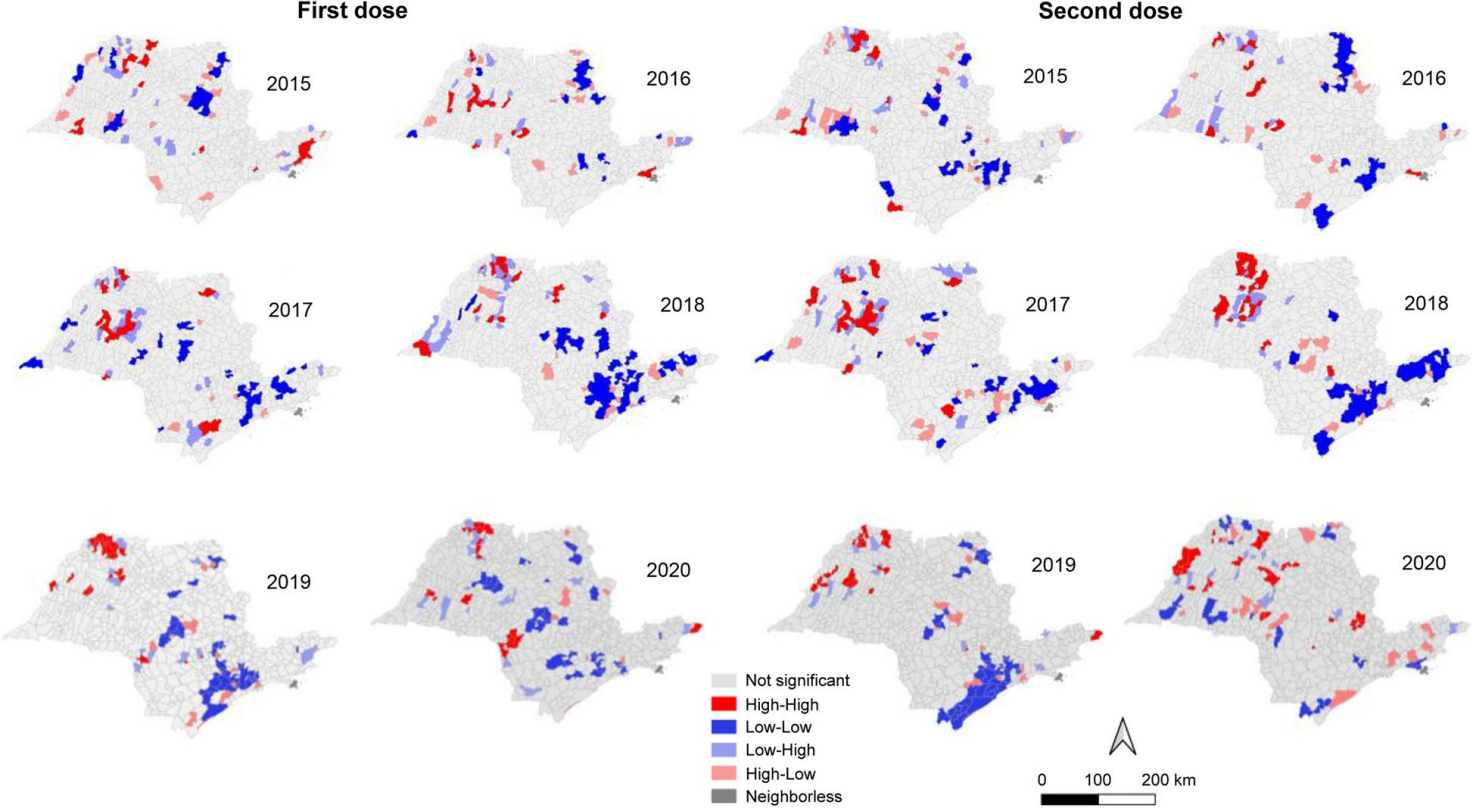
Moran’s map—spatial clusters for first and second dose measles vaccine coverage in the cities of the State of São Paulo from 2015 to 2020

A dynamic reading of the Moran map shows that the regions that had high rates of vaccination coverage, located in the high-high quadrant, in red, are static, i.e., they maintained a good measles vaccination coverage during the period of analysis and had a positive influence on their neighbors, more significantly on cities of the northwest region such as São José do Rio Preto, Catanduva, and Novo Horizonte, according to the administrative division of the State of São Paulo (Fig. 2).

However, the areas with low vaccination coverage (low-low quadrant), shown in blue, alternated each year, but the event was visibly more intense in the cities of the northern, São Paulo, and southern regions (Fig. 2). 

**Fig. 3:**
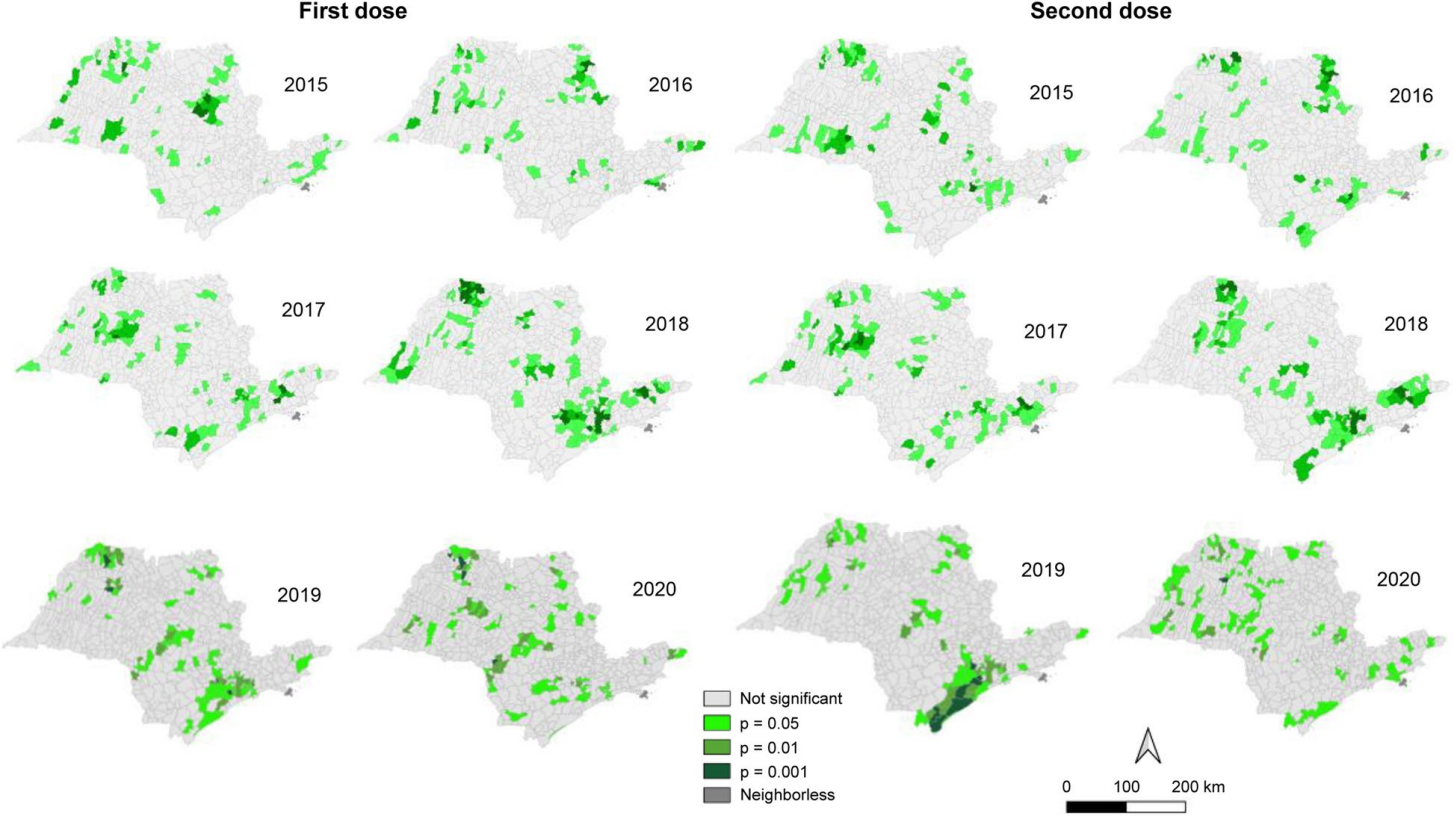
LISA map—areas with autocorrelation for first and second dose measles vaccine coverage in the cities of the State of São Paulo from 2015 to 2020

Figure 3 (LISA map) shows the areas with associative spatial patterns with greater significance for measles VC, reflecting a decomposition of the global association index.

Positive values were obtained in the GMI spatial autocorrelation test, i.e., we observed a dependence among the analysis units that influence the measles vaccination pattern in a dynamic way (Fig. 3).

## Discussion

The use of the spatial method associated with the Moran’s index was effective in this study, as it enabled the identification of priority areas to address the vaccination deficiency regarding to the recommended vaccination for PNI target to the measles vaccine in the State of São Paulo.

The temporal analysis of the spatial distribution of measles VC (Fig. 1) shows that coverage in the State of São Paulo is moderate in many cities and adequate in more than 80% of the state; however, second dose coverage was classified as low or very low also in more than 80% of the cities. This finding suggests that follow-up studies on adherence to measles vaccination should be conducted to generate evidence to explain this failure to comply with the regimen and enable the development of strategies to overcome missed vaccination.

The finding confirmed that there was an autocorrelation between the areas, and that these had a negative influence on their neighbors in terms of the pattern of vaccination coverage. Therefore, we infer that the cities located in the capital São Paulo and in the northern and northwestern regions form a group of priority areas for the intensification of measles vaccination activities, as they have the lowest rates of vaccination coverage and a negative influence on their neighbors.

In this study, a pattern of direct neighborhoods among units with low vaccination coverage was observed, indicating that the drop in measles vaccination coverage is somehow related to the geographic location and vaccine hesitance of these areas, thereby negatively influencing this index. Vaccination rates were more significant in areas with high population density, which according to the Brazilian Institute of Geography and Statistics are represented by Campinas, Guarulhos, São Paulo, São Bernardo do Campo, and São José dos Campos [20].

The geographic identification of population clusters with low vaccination coverage, as shown in some studies, allowed the identification of areas susceptible to outbreaks. These clusters emphasize the need to design strategies for continued measles eradication [21, 22]. The results of an analysis of VC in the five regions of the country, between 2006 and 2016, are in line with those obtained in the present study. The reduction in the triplet viral VC was up to 2.7% per year, with greater reductions in the most populated regions, an effect that we call proportional loss. However, the impacts of the spread of an infectious disease need to be seen in the same way. This implies that measures to recover vaccination rates need to be implemented, with priority in areas with the highest population density in the State of São Paulo [23].

Notably, the reduction in vaccination coverage has been a concern for the Ministry of Health since 2017; however, the advent of the pandemic worsened this scenario, as lack of vaccination among children is associated with the development of severe acute respiratory syndrome; in addition, H1N1 and Covid-19 co-infection has been reported [24]. Strategies must guarantee high and equal levels of vaccination coverage in the first and second doses, to reduce vaccine failures and assure the immunity of the population [20, 25]. Therefore, the identification of priority areas to address the low vaccination coverage is an essential measure for the control and eradication of MMR, and must be implemented by healthcare managers. According to Moura [26], it is important to implement strategies that strengthen the adherence to the PNI, whether regarding the demand or the increase in supply, including having alternative opening times in healthcare centers, mobile vaccination units, and campaign actions in important community settings, such as day care centers, schools, and churches.

In conclusion, it is necessary to develop strategies that guarantee that parents return with their children to the vaccination centers, because the spatial association was positive and more pronounced for the second dose. This finding confirms the observations of the need to complete the measles vaccination schedule, with reports that one dose is not effective in containing the spread of the infection [26].

The limitations related to this study are directly linked to the sources of health information. The immunization data was obtained from secondary and integrated information systems.

Another limitation was related to the data generated by the SI-PNI, which are neglected upfront due to the absence of a computerized system in many healthcare units [27, 28], thereby significantly delaying data updating, which should occur on a daily basis and in real time. This problem may prevent obtaining reliable results of vaccination coverage. It is noteworthy that even so, these systems are widely used in national and international research and that the data generated by them is essential for the planning of health activities in the country.

Although the results indicate that 'vaccine culture' is somehow associated with location and social characteristics [29], as this is a study with secondary data, it is not possible to make such a statement. Other factors such as the lack of infrastructure for the insertion of vaccine data by the health teams of the respective municipalities, added to other operational failures in the program itself, such as: lack of vaccine, lack of supplies, lack of health professionals, restricted opening hours, scheduling to avoid wasted doses can impact vaccine coverage.

## Conclusion

Based on the findings, we can conclude that the structure of immunization services is good throughout the State of São Paulo, as evidenced by the good vaccination coverage achieved with the first dose of the recommended measles regimen. However, the full distribution analysis of this coverage indicates that these services may present inefficiencies in complying with this scheme, as evidenced by the very low coverage rates for the second dose of the measles vaccine.

The authors emphasize the importance of conducting an on-site investigation to determine what factors are causing parents to fail to bring their children for the second vaccination dose. The authors also reinforce the importance of implementing strategic campaigns to educate parents and legal guardians on the importance of completing their children's vaccination schedules, in addition to the adoption of new health care models aimed at ensuring access to health services.

## Data Availability

We declare that the data used are from the public domain health and were obtained according to the criteria of good research practice and ethical precepts. The datasets analysed during the current study are available in the Zenodo Data repository, [https://doi.org/10.5281/zenodo.7374765].

## Abbreviations

GMI: Global Moran’s Index
IBGE: Brazilian Institute of Geography and Statistics
LISA: Local Indicators of Spatial Association
MMR: Measles, Mumps, and Rubella
PNI: National Immunization Program
SINASC: National Live Birth Information System
SUS: Unified Health System
VC: Vaccine coverage
